# Improving African Swine Fever Surveillance Using Fluorescent Rapid Tests

**DOI:** 10.3390/pathogens12060811

**Published:** 2023-06-07

**Authors:** Cristina Aira, Alejandro Monedero, Sonia Hernández-Antón, Juan Martínez-Cano, Ana Camuñas, Nadia Casado, Raquel Nieto, Carmina Gallardo, Marga García-Durán, Paloma Rueda, Alba Fresco-Taboada

**Affiliations:** 1 Gold Standard Diagnostics Madrid (GSD Madrid), Calle de los Hermanos García Noblejas 39, 28037 Madrid, Spain; 2 European Union Reference Laboratory for ASF, Centro de Investigación en Sanidad Animal (CISA-INIA/CSIC), Carretera Algete-El Casar de Talamanca, Km. 8.1, 28130 Madrid, Spain

**Keywords:** African swine fever, fluorescence, lateral flow assay, antigen detection, antibody detection, recombinant antibody, diagnostics

## Abstract

African swine fever (ASF) is a viral disease of swine with a huge impact due to its high mortality. Lately, the disease has actively spread around the world, affecting new areas from which it had been eradicated long ago. To date, ASF control is carried out by the implementation of strict biosecurity measures such as the early identification of infected animals. In this work, two fluorescent rapid tests were developed to improve the sensitivity of point-of-care diagnosis of ASF. For antigen (Ag) detection in blood, a double-antibody sandwich fluorescent lateral flow assay (LFA) was developed, employing a newly developed recombinant antibody to the VP72 of the virus. To complement the diagnosis, a double-recognition fluorescent LFA was developed using the VP72 for the detection of specific antibodies (Ab) in sera or blood. Both assays statistically improved the detection of the disease when compared to the commercial colorimetric assays INgezim^®^ ASFV CROM Ag and INgezim^®^ PPA CROM Anticuerpo, respectively, with higher statistical significance between 11 and 39 days post-infection. From the observation of results, it can be concluded that the combination of both Ag-LFA and Ab-LFA assays would facilitate the identification of infected animals, regardless of post-infection time.

## 1. Introduction

African swine fever (ASF) is a contagious infectious disease of domestic pigs and wild boar with a high impact on the animal’s wellbeing and on the swine industry. Infected animals can present a wide range of clinical syndromes depending on the viral strain’s virulence and host characteristics. In clinical terms, several forms can be differentiated: an acute form of the disease, which correlates with hemorrhagic fever and typical ASF symptoms with high fatality rates (up to 95–100%); a subacute form, which presents similar, although less severe, signs to those observed in acute forms and exhibits mortality rates of 30–70%; and a chronic form of the disease, with unspecific symptoms which last for months and do not resemble the typical ASF picture [1,2,3].

The disease was first described in Kenya in 1921, from where it spread to Europe and America during the mid-20th century. Although it was successfully eradicated from most of the affected countries through strict control measures, ever since, it has been endemic to Africa and Sardinia. In 2007, the disease was reintroduced in Eastern Europe. Afterwards, ASFV spread to China in 2018, affecting the entire Asian continent and the Indo-Pacific region within a few years. Recently (2021–2023), the disease was also notified in countries from where it had been eradicated a long time ago such as Haiti, the Dominican Republic, Germany, Italy, and Greece [3,4,5,6]. Due to its high socioeconomic impact and its transboundary threat, ASF is a notifiable disease to the World Organization for Animal Health (WOAH) [5,7].

ASF is caused by infection with ASF virus (ASFV), a large multienveloped double-stranded DNA virus, classified as the only member of the *Asfarviridae* family within the *Asfivirus* genus [8,9]. It is known that ASFV has a complex structure composed of several capsids and lipid membranes; however, detailed knowledge on the virion structure is still needed. To date, up to 54 different structural proteins have been described [7,10,11,12].

The ASFV genome is highly conserved; it encodes more than 150 open reading frames, and the main differences in genome length are due to tandem repeats and the gain or loss of multigene family (MGF) copies. A sequence comparison of the B646L gene (codifying for p72) demonstrated that this gene is highly conserved among ASFV strains isolated from different parts of the world, and led to the definition of 24 viral genotypes following WOAH-recommended diagnostic PCR and phylogenetic analysis, mainly focused in the C-terminus of the protein [13,14]. The p72 protein, together with other major components of the viral capsid (p30, p54, and the polyprotein pp62), have been described as the most antigenic proteins. Among them, the p72 is the main component of the outer capsid, including 8280 copies of this protein, and it is estimated that this protein involves around one third of the ASFV virion [5,10]. Due to its high conservation (97.8–100% amino acid sequence identity), its high presence in virion composition, and its immunogenicity, p72 has long been considered an important antigen for ASF diagnosis [10,13].

Despite the resources put into place for decades, a safe and secure commercial vaccine for ASFV control is not yet available despite the on-farm trials of vaccines in Vietnam, with one of them expected to be commercialized shortly [15,16]. ASF control is still based on early diagnosis and on the enforcement of strict sanitary measures. For this reason, access to highly sensitive diagnostic tests is crucial to control the disease. The combination of direct antigen detection together with the detection of specific antibody methods is particularly relevant for ASF monitoring. As defined by WOAH, virus detection can be performed through virus isolation, fluorescent antibody tests, PCR, or double-antibody sandwich ELISA. To investigate the serological status, ELISA, indirect immunoperoxidase tests, indirect fluorescent antibody tests, and immunoblotting are recommended [17,18].

All the above-described methods need trained personnel and must be performed in a lab. On the contrary, lateral flow assays (LFAs) are user-friendly, low-cost, give rapid results, and exhibit long-term stability over a wide range of climates, making them one of the most widely used techniques for point-of-care testing [19]. Despite these advantages, LFAs still lack enough sensitivity to meet the performance of reference laboratory assays, being recommended only as an outbreak investigation method and for the routine testing of sick animals [20]. To improve the implementation of LFAs, new fluorescent labels have been developed to increase the overall sensitivity. Among them, lanthanide chelate-doped polystyrene nanoparticles have gained attention due to their higher fluorescence than free dye molecules, low variation, and long-lifetime fluorescence, also providing the advantages of common colored polystyrene nanoparticles [21,22,23,24].

LFA technology for antigen detection usually relies on the use of specific monoclonal antibodies (MAbs). The recombinant MAb (rMAb) methodology allows the higher characterization of these molecules, enabling more reproducible batches and the possibility of protein engineering [25,26].

In this work, we describe the optimization of ASFV detection through the implementation of highly sensitive fluorescent rapid tests employing europium-labelled nanoparticles for the direct detection of the virus and for ASF surveillance through the detection of specific antibodies.

## 2. Materials and Methods

### 2.1. Blood and Serum Samples

For antigen detection, a collection of 56 blood samples collected from 54 domestic pigs at different days post-infection from previous experimental studies, and kept at −80 °C until their use, were evaluated. Animals were inoculated with different ASFV strains, including genotypes I (*n* = 11), II, (*n* = 42), I–II (*n* = 1), IX (*n* = 1), and XXIII (*n* = 1). The samples were characterized as positive using WOAH real-time PCR and they were evaluated in parallel with the commercial LFA INgezim^®^ ASFV CROM Ag (GSD Madrid, Madrid, Spain). To evaluate assay specificity, a total of 100 field blood samples collected in ASFV-free areas from domestic pigs were evaluated.

For antibody detection, a collection of 141 serum samples and 28 blood samples, collected from 128 domestic pigs during the same experimental studies and kept at −80 °C until their use, were evaluated. Animals were inoculated with different ASFV strains, including genotypes I (*n* = 60), II (*n* = 102), I–II (*n* = 2), X (*n* = 2), and XXIII (*n* = 3). The samples were characterized as positive using an immunoperoxidase test (IPT) and they were also analyzed with the commercial competitive ELISA INgezim^®^ PPA COMPAC as well as with the LFA INgezim^®^ PPA CROM Anticuerpo (GSD Madrid, Madrid, Spain). To evaluate assay specificity, a collection composed of 34 field blood samples collected from domestic pigs from ASFV-free regions, 40 negative experimental sera, 10 negative field sera, 10 sera positive for *Mycobacterium tuberculosis*-specific antibodies, 10 sera positive for porcine-respiratory-and-reproductive-virus-specific antibodies, and 10 sera positive for classical-swine-fever-virus-specific antibodies was evaluated.

### 2.2. Generation of the Recombinant Monoclonal Antibody (rMAb)18BG3 from the Hybridoma Cells

The raw material for the construction of the rMAb was a specific hybridoma against ASF VP72, which had been previously generated and used as a regular source of 18BG3 MAb. The total RNA was isolated from 1 × 106 hybridoma cells (PureLink™ RNA Mini Kit, Invitrogen, Waltham, MA, USA) and quantified. A total of 30 ng of RNA were used as a template in a one-step RT-PCR performed with the enzyme SuperScript III (Invitrogen, Waltham, MA, USA). The properly degenerated primers were used in mixes to hybridize to the light and heavy chains of the MAb. The RT-PCR products were sequenced, and the variable regions were defined by homology with databases from NCBI BLAST and NCBI Conserved Domain Search. Specific primers were designed to hybridize the delimitated sequences and to include restriction sites in the 5′-end of the variable heavy chain (VH) (HindIII) and 3′-end of the variable light chain (VL) (Xho I). In addition, the DNA complementary sequences corresponding to the peptide linker (G4S) × 3 were added in 3′VH and 5′VL. Two independent PCRs were performed with those primers using VH and VL cDNAs as templates. Both PCRs were performed with the FastStart Taq polymerase (Roche, Basel, Switzerland) according to the manufacturer setting conditions. Finally, the resulting amplified products were employed in an overlap extension PCR to assemble the single-chain variable fragment (scFv) construct connecting 3′VH to 5′VL through the linker. This scFv was cloned by HindIII/Xho I digestion and subsequently ligated into the expression vector pCMV6-AC-Fc-S (Origene, Rockville, MD, USA) that adds a mouse Fc tag at the 3′-end of the cloned sequence. Hence, the final format of the rMAb was a scFv-Fc.

The cloned sequence was corroborated using automatic sequencing (Eurofins Genomics, Ebersberg, Germany), and the plasmid was purified (Qiagen plasmid plus kit, Qiagen, Germantown, MD, USA) and transfected in suspension cultures of HEK293 FreeStyle cells (Invitrogen, Waltham, MA, USA). The DNA was added at 1 µg of plasmid/mL of cell culture using the cationic polymer FectoPro (Polyplus™, Illkirch-Graffenstaden, France). After four days at 37 °C, 5% CO_2_, and 125 rpm, the culture was collected, the supernatant separated with centrifugation, and the rMAb purified from the supernatant with affinity chromatography to protein A (mAb Select, Cytiva, Marlborough, MA, USA). The recombinant antibody was analyzed using SDS-PAGE and an indirect ELISA to assess its purity and activity, respectively.

### 2.3. Fluorescent Lateral Flow Assays for Antigen Detection of ASFV (Ag-LFA) or Antibody Detection (Ab-LFA)

For the detection of antigen in blood samples, a double-antibody sandwich assay was developed.

#### 2.3.1. Capture Reagents

For the development of the antigen LFA (Ag-LFA), the commercial monoclonal antibody (MAb) 18BG3 (GSD Madrid, Madrid, Spain) specific to the p72 of ASFV was used as a capture reagent. It was diluted to 0.4 mg/mL in 20 mM Tris-HCl pH 7.5 buffer containing 5% (*w*/*v*) sucrose and it was dispensed at 1 µL/cm on a nitrocellulose membrane as test line using a Biodot XYZ3060 platform.

For the development of the antibody LFA (Ab-LFA), semipurified p72 was diluted in Tris-HCl pH 8.5 containing sucrose and the solution was dispensed at 1 µL/cm onto a nitrocellulose membrane as a capture reagent using the same platform.

As a control line capture reagent, a specific monoclonal antibody to biotin diluted to 0.1 mg/mL in the same buffer was dispensed in parallel to the test line in both assays. The resulting membrane was dried for 5 min at 45 °C and stored at room temperature under dry conditions.

#### 2.3.2. Detector Reagents

The 18BG3 rMAb, the VP72, and the control protein (BSA labelled with biotin) were covalently coupled to carboxylated polystyrene latex nanoparticles internally labelled with europium (ThermoFisher Scientific, Waltham, MA, USA). Briefly, carboxyl groups on the surface of the nanoparticles were activated according to the EDC/NHS protocol [27] based on a two-step carbodiimide reaction. Then, activated nanoparticles were separately incubated with the different reagents (18BG3 rMAb, VP72, and control protein, respectively), which were added to obtain a final concentration of 1 mg/m^2^. After blocking the nonreacted functional groups, the particles were diluted to 1% (*w*/*v*) in 10 mM Tris-HCl pH 8.2 and they were stored at 4 °C in the dark.

To prepare the conjugate solutions, for Ag-LFA 18BG3 rMAb europium latex was mixed with control europium latex diluted to a final concentration of 0.01% (*w*/*v*) and 0.005% (*w*/*v*), respectively, and the mixture was dispensed onto the conjugate pad. For Ab-LFA, latex beads coupled to p72 were mixed with control system particles to a final concentration of 0.01% (*w*/*v*) and 0.005% (*w*/*v*), respectively, and dispensed onto a conjugate pad. The pads were dried overnight at room temperature and stored at room temperature under dry conditions.

#### 2.3.3. Assembling of LFA Strips

The nitrocellulose membrane was pasted on an adhesive card (backing card), followed by conjugate and absorbent pads, both overlapping the nitrocellulose membrane. The sample pad was pasted on the backing card overlapping the conjugate pad and, finally, an adhesive cover tape was pasted onto all the materials, leaving only a small application area on the sample pad. This master card was cut into 4.2 mm width strips, and individual strips were assembled into cassettes and stored at room temperature in aluminum foil under dry conditions (Supplementary Figure S1).

#### 2.3.4. Test Procedure

The Ag-LFA was developed to be used with blood samples. Bloods analyzed in the present work were conserved at 2–8 °C for up to 15 days or frozen at −80 °C. To perform the test, 20 µL of whole blood was added to the sample application window. After waiting 1 min for the blood to filtrate, 3 drops of running buffer (Tris-HCl, pH 7.5, containing Tween20, NaCl, casein, and NaN_3_) were added one by one to the buffer addition window. Assays were incubated at room temperature for 15 min and interpretation of the assay result was performed with a UV reader (Pacific Image Electronics, New Taipei City, Taiwan) to obtain a quantitative value.

The Ab-LFA was developed to be used with blood or serum samples. Bloods analyzed in this study were refrigerated at 2–8 °C or frozen at −80 °C. Sera analyzed in this study were frozen at −20 °C or −80 °C. To perform the assay, 20 µL of blood or 10 µL of serum was added to the cassette window and, after waiting 1 min for sample filtration, 3 drops of running buffer (Tris-HCl, pH 7.5, containing Tween20, NaCl, casein, urea, and NaN_3_) were added one by one. The assays were incubated at room temperature for 15 min and the results were read out as described for Ag-LFA.

### 2.4. Statistical Analysis

A sufficient number of samples were evaluated to demonstrate the proof of principle of the described assays. We calculated 95% CIs for ASFV samples classified according to days post-infection. For the statistical significance determination between the commercial colorimetric assays and the newly developed fluorescent assays, a McNemar test was performed using the open access software OpenEpi 3.01 (Open Source Epidemiologic Statistics for Public Health, Online Version: www.OpenEpi.com. © 2003 Atlanta, GA, USA).

For specificity calculation, samples that gave a negative signal with the reference technique but positive with the developed assay were considered false positives, while concordant negative results were considered true negatives.

(1) $$\mathrm{Specificity}=\frac{\mathrm{True}\;\mathrm{negatives}}{\mathrm{True}\;\mathrm{negatives}+\mathrm{False}\;\mathrm{positives}}$$

## 3. Results

### 3.1. Recombinant Antibody Production

**Figure 1:**
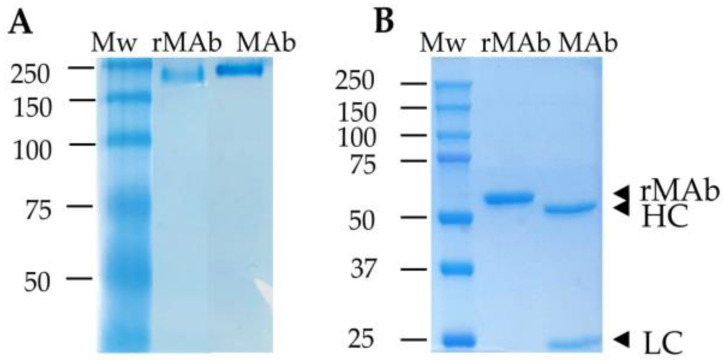
Coomassie staining of polyacrylamide gels loaded with 2 µg of rMAb and MAb in native (**A**) or denaturing conditions (**B**). Mw: molecular weight markers; HC: heavy chain; LC: light chain.

The sequence of the variable regions of 18BG3 was amplified from RNA. The sequence of scFv was correctly assembled and cloned in the mammalian expression vector pCMV6-AC-S-Fc. The purified plasmid containing the expected sequence was transfected in suspension cultures of HEK293 cells and the cultures were collected on the fourth day. The rMAb purified from the supernatant yielded around 50 mg per liter of medium and showed a high degree of purification in the polyacrylamide gel electrophoresis analysis. The native antibody (Mab) and the recombinant antibody (rMAb) were compared through Coomassie staining; in denaturing conditions, the two chains of the Mab (heavy and light) were separated and detected independently, but the rMAb had only one chain as heavy and light chains are expressed together (Figure 1). The specificity of the rMAb was assessed in an indirect ELISA assay and the MAb was included as the control, showing similar performance.

### 3.2. Fluorescent Double-Antibody Sandwich LFA for Detection of ASFV Antigen, Ag-LFA

#### 3.2.1. Analytical Sensitivity

**Figure 2:**
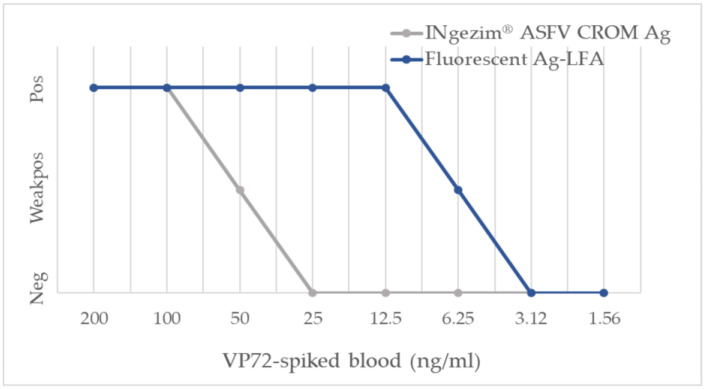
Comparison of analytical sensitivity of the new fluorescent assay to the commercial assay INgezim^®^ ASFV CROM Ag.

First, the analytical performance of the new fluorescent assay was evaluated. For this purpose, VP72 was serially diluted in assay buffer, obtaining an analytical sensitivity of 250 pg/test. When the same titration of VP72 was prepared on negative blood matrix, a higher sensitivity was observed of 125 pg/test, indicating that there was no interference from the matrix in antigen detection. Last, the analytical sensitivity of the new assay was compared to the commercial assay INgezim^®^ ASFV CROM Ag (GSD Madrid, Madrid, Spain). As shown in Figure 2, the use of fluorescent particles increased the sensitivity of the assay 8-fold, passing from a limit of detection of 50 ng/mL of VP72 in negative blood to 6.25 ng/mL. The results were confirmed by triplicates for all the calculations shown.

#### 3.2.2. Diagnostic Performance

**Figure 3:**
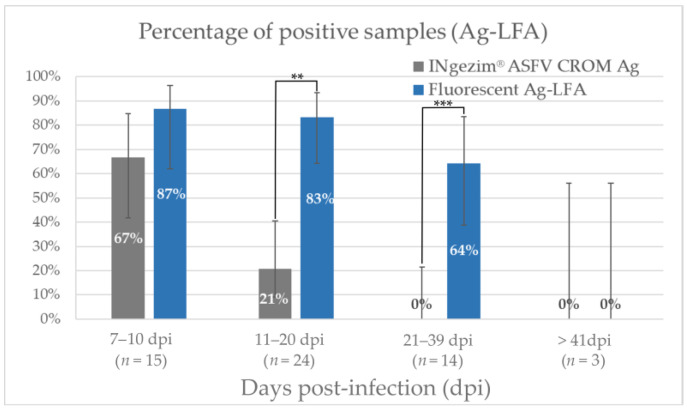
Percentage of positive samples with the commercial assay INgezim^®^ ASFV CROM Ag and with the new fluorescent Ag-LFA in the different groups divided according to days post-infection. Bars show the 95% confidence interval for each group. ** *p* value < 0.05. *** *p* value < 0.01.

To evaluate the performance of the assay, 56 positive experimental samples were analyzed. As shown in Figure 3, the fluorescent assay significantly improved antigen detection when compared to the commercial colorimetric assay. In the group of samples collected between 7 and 10 days post-infection (dpi) (*n* = 15), the fluorescent assay detected 87% of the samples as positive while the commercial colorimetric assay detected 67%. No statistical significance was obtained in that group (*p* = 0.13), although a trend can be observed in the data. In the group of samples collected between 11 and 20 dpi (*n* = 24), a statistically significant increment in the assay’s sensitivity was observed, passing from 21% of positive samples to 83% (*p* = 0.04). In the group of 21–39 dpi (*n* = 14), 64% of the samples were identified as positive with the fluorescent assay, while no positive samples were detected with the commercial colorimetric assay, exhibiting a high statistical significance (*p* = 0.004). No positive samples were detected after 41 dpi (*n* = 3) with the fluorescent assay or with the commercial INgezim^®^ ASFV CROM. This might be explained because viral load in the blood decreases after a long time post-infection.

For the evaluation of assay specificity, a total of 100 negative blood samples collected from ASF-free areas were evaluated using both assays (Table 1). Only 2 out of the 100 samples analyzed gave a false-positive result, yielding a specificity of 98%.

### 3.3. Fluorescent Double Recognition LFA for Detection of ASFV p72-Specific Antibodies

#### 3.3.1. Analytical Performance

**Figure 4:**
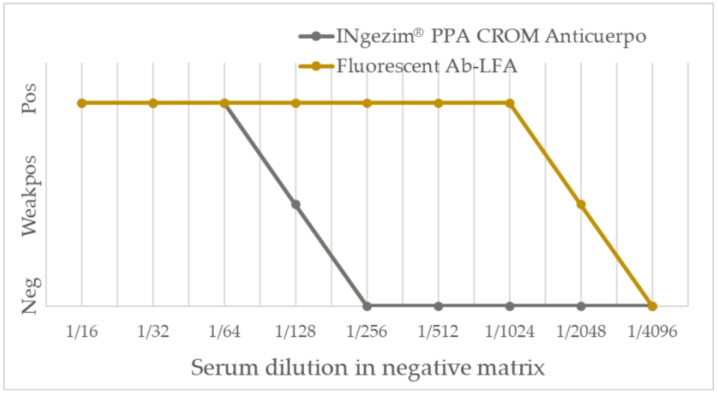
Comparison of analytical sensitivity of the new fluorescent assay to the commercial assay INgezim^®^ PPA CROM Anticuerpo.

Once the best conditions for antibody detection assay were set, the analytical sensitivity was determined. For that purpose, the anti-VP72 MAb-18BG3 was diluted in assay buffer obtaining a limit of detection of 90 pg/test. No matrix interference was observed when the antibody dilution was performed in negative serum, obtaining a limit of detection of 62 ng/test, a similar limit of detection as the one observed only with buffer. Last, the analytical sensitivity of the new fluorescent assay was compared to that obtained with the commercial assay INgezim^®^ PPA CROM Anticuerpo. A positive hyperimmune serum prepared at the CISA-INIA/CSIC through experimental infection was serially diluted in negative serum and analyzed with both assays. As shown in Figure 4, the fluorescent assay detected the sample as positive down to the 1/2048 dilution, whereas the commercial assay detected the sample as positive only down to the 1/128 dilution. Thus, the use of fluorescent particles increased the analytical sensitivity of the assay sixteen-fold. The results were confirmed by triplicates for all the calculations shown.

#### 3.3.2. Diagnostic Performance

The diagnostic performance of the assay was evaluated with 169 experimental positive serum and blood samples and 114 negative serum and blood samples.

**Figure 5:**
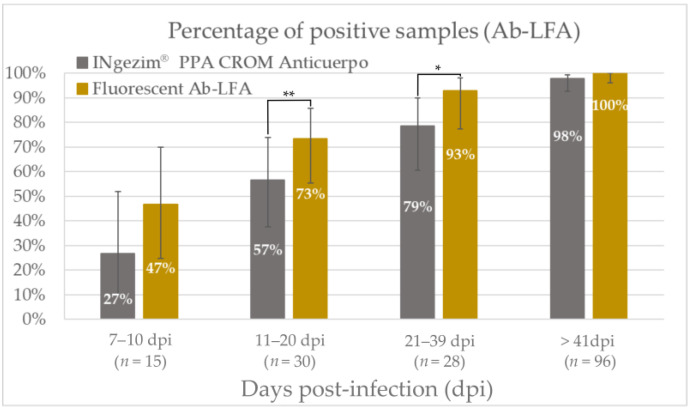
Percentage of positive samples with the commercial assay INgezim^®^ PPA CROM Anticuerpo and with the new fluorescent Ab-LFA in the different groups divided according to days post-infection. Bars show the 95% confidence interval for each group. ** *p* value < 0.05. * *p* value < 0.1.

As shown in Figure 5 and stated in Table 2, the fluorescent assay improved the antibody detection in all the groups tested. Within the group of 7 to 10 dpi (*n* = 15), the percentage of samples detected as positive increased from 27% with the commercial rapid test to the 47% obtained with the fluorescent assay; there was no statistical significance in this group (*p* = 0.13). In the group of 11 to 20 dpi (*n* = 30), the percentage increased from 57% to 73%, showing a statistical significance greater than 95% (*p* = 0.04). Furthermore, in the group of 21 to 39 dpi (*n* = 28), the percentage increased from 79% to 93%, with a statistical significance higher than 90% (*p* = 0.07). A lower improvement in assay sensitivity was observed in the group of more than 41 dpi (*n* = 96), where antibody titers were higher and both assays had high percentages of positive samples (*p* = 0.24).

For the evaluation of diagnostic specificity, field and experimental negative samples were evaluated (Table 2). No false-positive results were obtained among the 50 negative sera nor among the 34 negative bloods. Moreover, 30 sera positive for other related diseases were tested. No cross-reaction was observed with the sera positive for antibodies against classical swine fever virus, tuberculosis, or porcine reproductive and respiratory virus. The new fluorescent Ab-LFA exhibited a specificity of 100%.

## 4. Discussion

African swine fever is one of the most complex and misunderstood infectious diseases of swine, which causes great concern in the pig industry because of its massive socioeconomic impacts. During the last few years, the virus has spread fast through Asia, the Indo-Pacific region, Europe, and Central America, recently affecting countries from where the disease had been eradicated long ago, such as Italy, Germany, or the Dominican Republic [4,6,28]. Since no treatment or licensed vaccines are available yet, the rapid and reliable detection of this disease is crucial for the proper implementation of biosecurity measures that allow the control of the pandemic. Clinical signs can vary depending on animal and virus characteristics, and infection by ASFV can be confused with other diseases. For that reason, only a laboratory diagnosis can properly identify animals that are infected or have previously been infected with the virus [28,29,30]. Considering that, rapid tests can help to control ASFV spread through the fast on-site identification of infected animals. In this work, the biggest limitation of rapid tests, their sensitivity, has been addressed using fluorescent nanoparticles, which has shown to improve the limit of detection of diagnostic assays [24,31,32].

Antigen detection is a direct indicator of infection, and it is crucial for the detection of infected animals [33]. For the direct detection of ASFV in blood samples, the new fluorescent assay improved the early detection of the infected animals, detecting 87% of positive samples in the group of 7–10 dpi and 83% in the group of 11–20 dpi. Moreover, this new fluorescent assay allowed the detection of positive samples on later days post-infection, showing a percentage of positive samples of 64% in the group of 21–38 dpi, where no positive samples were detected with the colorimetric assay (Figure 3). Thus, the fluorescent assay statistically improved the viral detection of ASFV in blood samples. On the other hand, the presence of antibodies to ASFV indicates a current or past exposure to the virus since no massive vaccination has been carried out to date [29]. In this work, the use of fluorescent particles improved the analytical sensitivity of the colorimetric assay for antibody detection by sixteen times. As shown in Figure 5, this sensitivity improvement allowed the earlier detection of antibodies in experimental samples, detecting 47% of positive samples between 7 and 10 dpi, and increasing the positive samples detected in all the groups analyzed.

The combined use of antigen detection and antibody detection tests in parallel has the potential to improve the diagnosis of African swine fever, since when the detected antigen levels with the Ag-LFA decreased as the days post-infection developed (Figure 3), the samples positive to antibodies detected by Ab-LFA increased (Figure 5 and Figure S2). Then, the combination of antigen and antibody detection would allow the detection of near to 100% of the animals which are or have been affected by ASFV, which is of special interest since not only acute-infected animals, but also carrier swine and persistently infected wild pigs, require special consideration in controlling the disease [17,18].

## Figures and Tables

**Table 1:** Results obtained with the samples evaluated with the commercial INgezim^®^ ASFV CROM Ag and with the new fluorescent Ag-LFA.

	Group of Samples	Result from INgezim^®^ ASFV CROM Ag (No. of Samples)		Result from Fluorescent Ag-LFA (No. of Samples)	
		Positive	Negative	Positive	Negative
Positive samples (*n* = 56)	7–10 dpi	10	5	13	2
	11–20 dpi	5	19	20	4
	21–39 dpi	0	14	9	5
	>41 dpi	0	3	0	3
Negative samples (*n* = 100)	Field bloods	2	98	2	98

**Table 2:** Results obtained with the samples evaluated with the commercial INgezim^®^ PPA CROM Anticuerpo and with the new fluorescent Ab-LFA.

	Group of Samples	Result from INgezim^®^ PPA CROM Anticuerpo (No. of Samples)		Result from Fluorescent Ab-LFA (No. of Samples)	
		Positive	Negative	Positive	Negative
Positive samples (*n* = 141)	7–10 dpi	4	11	7	8
	11–20 dpi	17	13	22	8
	21–39 dpi	22	6	26	2
	> 41 dpi	94	2	96	0
Negative samples (*n* = 114)	Field sera	0	10	0	10
	Experimental sera	0	40	0	40
	Field bloods	0	34	0	34
	CSFV-positive sera	0	10	0	10
	TB-positive sera	0	10	0	10
	PRRSV-positive sera	0	10	0	10

## Data Availability

The data presented in this study are available on request from the corresponding author.

## Supplementary Materials

The following supporting information can be downloaded at: https://www.mdpi.com/article/10.3390/pathogens12060811/s1. Figure S1: Scheme of the different components of the lateral flow tests. A. Fluorescent antigen test (Ag-LFA). B. Fluorescent antibody test (Ab-LFA). Figure S2: Percentage of positive samples with the new fluorescent assays: Ag-LFA and Ab-LFA in the different groups divided according to days post-infection. Bars show the 95% confidence interval for each group. Samples analyzed with Ag-LFA and Ab-LFA shown in the figure are not the same for both assays.
