# Supplementary material for: Improving African Swine Fever Surveillance Using Fluorescent Rapid Tests
Source: Pathogens. 2023 Jun 7;12(6):811. doi: 10.3390/pathogens12060811 (PMC10303979; doi:10.3390/pathogens12060811)
Supplement: Supplementary file 1 [file pathogens-12-00811-s001.zip › pathogens-2419346-supplementary.pdf]

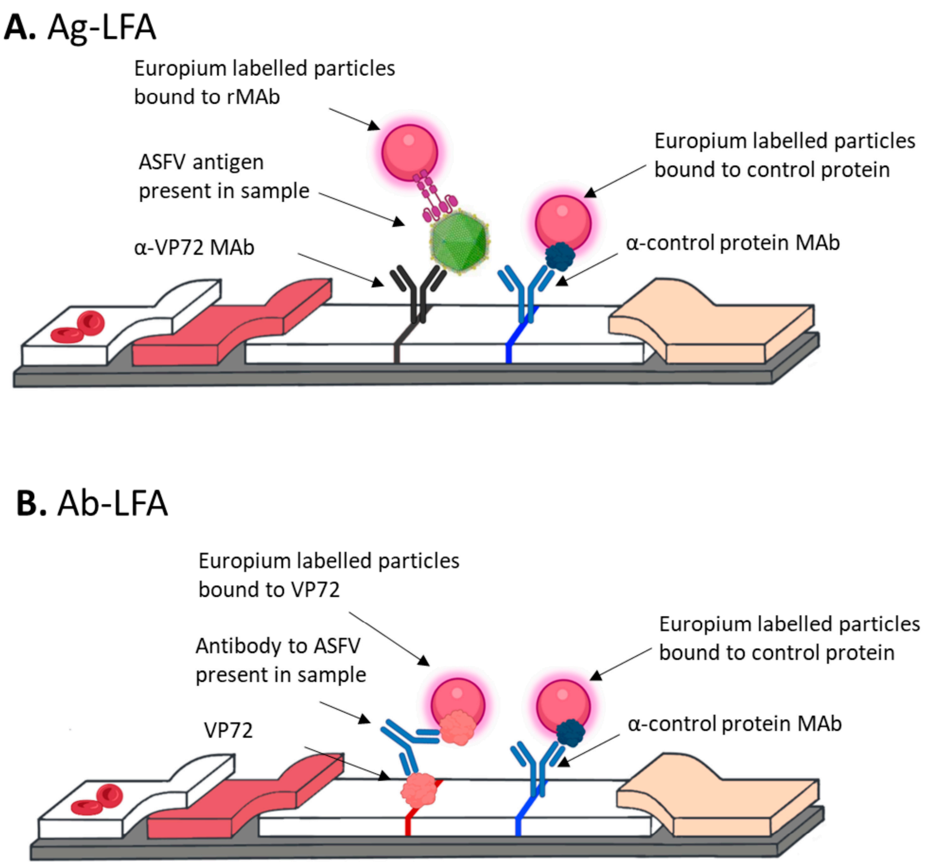

Figure S1. Scheme of the different components of the lateral flow tests. A. Fluorescent antigen test (Ag-LFA). B. Fluorescent antibody test (Ab-LFA).

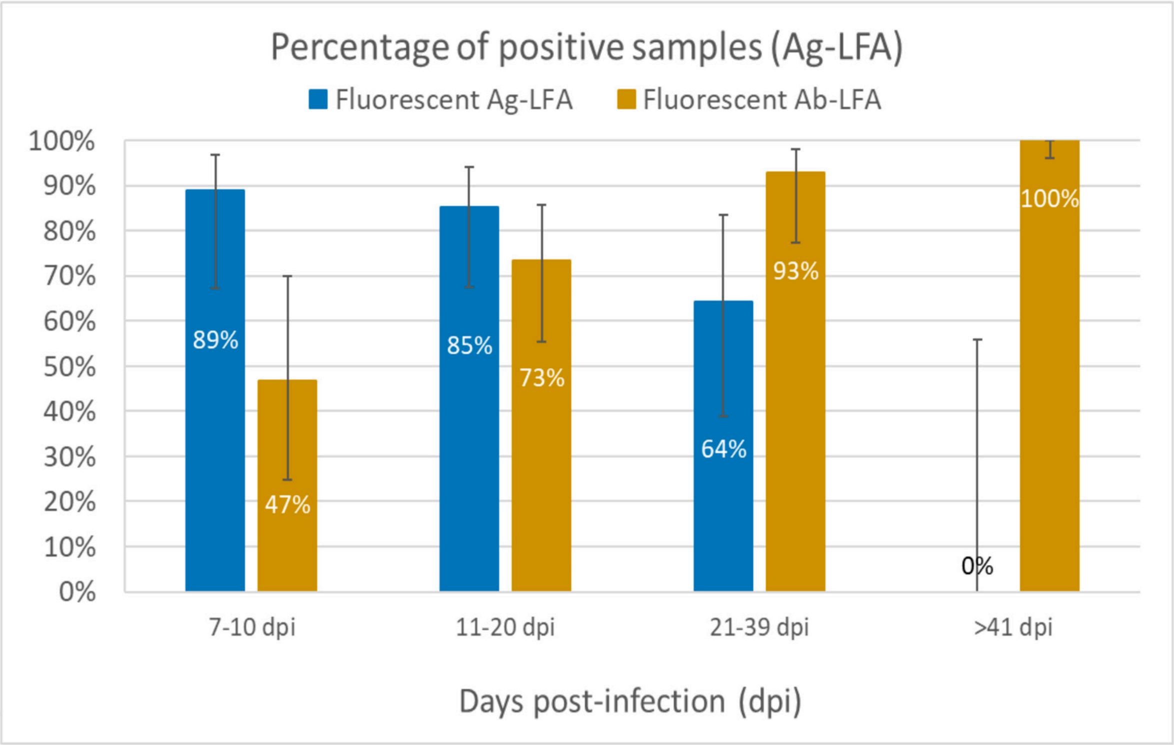

Figure S2. Percentage of positive samples with the new fluorescent assays: Ag-LFA and Ab-LFA in the different groups divided according to days post-infection. Bars show the 95% confidence interval for each group. Samples analyzed with Ag-LFA and Ab-LFA shown in the figure are not the same for both assays.
